# Extracellular Localisation of the C-Terminus of DDX4 Confirmed by Immunocytochemistry and Fluorescence-Activated Cell Sorting

**DOI:** 10.3390/cells8060578

**Published:** 2019-06-12

**Authors:** Yvonne L. Clarkson, Emma Weatherall, Martin Waterfall, Marie McLaughlin, Haojiang Lu, Paul A. Skehel, Richard A. Anderson, Evelyn E. Telfer

**Affiliations:** 1 Institute of Cell Biology, University of Edinburgh, Edinburgh EH9 3FF, UK; eweatherall@moredun-scientific.com (E.W.); m.mclaughlin@ed.ac.uk (M.M.); haojiang.lu@ki.se (H.L.); evelyn.telfer@ed.ac.uk (E.E.T.); 2 Centre for Discovery Brain Sciences, University of Edinburgh, Edinburgh EH8 9XD, UK; paul.skehel@ed.ac.uk; 3 Ashworth Laboratories, School of Biological Sciences, University of Edinburgh, Edinburgh EH9 3JR, UK; Martin.Waterfall@ed.ac.uk; 4 MRC Centre for Reproductive Health, Queens Medical Research Institute, University of Edinburgh, Edinburgh EH16 4TJ, UK; richard.anderson@ed.ac.uk

**Keywords:** ovarian stem cells, DDX4, fluorescence-activated cell sorting, immunocytochemistry, human

## Abstract

Putative oogonial stem cells (OSCs) have been isolated by fluorescence-activated cell sorting (FACS) from adult human ovarian tissue using an antibody against DEAD-box helicase 4 (DDX4). DDX4 has been reported to be germ cell specific within the gonads and localised intracellularly. White et al. (2012) hypothesised that the C-terminus of DDX4 is localised on the surface of putative OSCs but is internalised during the process of oogenesis. This hypothesis is controversial since it is assumed that RNA helicases function intracellularly with no extracellular expression. To determine whether the C-terminus of DDX4 could be expressed on the cell surface, we generated a novel expression construct to express full-length DDX4 as a DsRed2 fusion protein with unique C- and N-terminal epitope tags. DDX4 and the C-terminal myc tag were detected at the cell surface by immunocytochemistry and FACS of non-permeabilised human embryonic kidney HEK 293T cells transfected with the DDX4 construct. *DDX4* mRNA expression was detected in the DDX4-positive sorted cells by RT-PCR. This study clearly demonstrates that the C-terminus of DDX4 can be expressed on the cell surface despite its lack of a conventional membrane-targeting or secretory sequence. These results validate the use of antibody-based FACS to isolate DDX4-positive putative OSCs.

## 1. Introduction

The field of reproductive biology is divided over the possibility of neo-oogenesis in the adult mammalian ovary [1]. The scientific dogma, held for over fifty years, is that mammalian neo-oogenesis does not continue in adult life for the majority of mammals, including humans, and hence addition to the primordial follicle pool formed before or shortly after birth is not possible [2]. In 2004, Tilly and colleagues challenged this [3], and subsequent data have also provided evidence for the existence of a population of putative oogonial stem cells (OSCs) in mice [4,5,6,7,8,9,10,11,12,13], rats [14], cattle [15] and humans [6,15,16,17,18,19,20,21,22,23,24]. These have the potential to form functional oocytes both in vitro [4,5,6,7,8,14,16,17,19,20,21,25] and in vivo [6,14] although as yet no physiological role has been demonstrated.

A key factor in the isolation of putative OSCs from mouse and human ovaries is the use of an antibody against the C-terminus of the germline marker Ddx4/DDX4. This was used in conjunction with magnetic-activated cell sorting (MACS) and fluorescence-activated cell sorting (FACS) to isolate Ddx4/DDX4-positive populations [4,6,7,15,16,20,25,26,27,28,29,30]. However, this approach relied on the presence of an extracellular DEAD-box helicase 4 (DDX4) epitope that would not be predicted from its primary structure. Human *DDX4*, a homologue of the highly conserved *VASA* gene, encodes an ATPase protein with RNA helicase activity. It is expressed in the germ cell lineage in males and females and functions in germ cell development [31]. However, as an RNA helicase, DDX4 would be anticipated to be an exclusively intracellular protein [32,33,34], challenging the DDX4 expression model (Figure S1) proposed by White and colleagues [6], wherein DDX4/Ddx4 protein is present on the surface of OSCs, and subsequently internalised during the process of oogenesis. The DDX4-positive cell populations isolated by White and colleagues using FACS formed oocyte-like structures in culture suggesting putative OSCs had been isolated. Notably, using an antibody against the N-terminus of DDX4, no DDX4-positive cells could be isolated unless the cells were permeabilised, suggesting that the C-terminus of DDX4 is extracellular, while the N-terminus is intracellular.

Several groups have published reports stating or showing that Ddx4/DDX4-positive cells cannot be isolated using these cell sorting methods [26,35,36]. Hernandez and colleagues [26] created a lentivirus encoding a fusion protein to detect the C-terminus of DDX4 indirectly by tagging it with a myc epitope, so for the first time DDX4 detection was not reliant on the C-terminus DDX4 antibody. In live transduced human ovarian cells, the antibody against the C-terminus of DDX4 was highly expressed but there was no expression of the myc tag, suggesting a high degree of non-specificity of the C-terminus antibody.

In order to address these inconsistencies, the aim of this study was to use molecular tools to determine whether localisation of the C-terminus of human DDX4 on the cell surface was possible. A novel construct, pFLAG-DDX4-myc, was generated to express full-length human DDX4 with an N-terminal FLAG epitope and a C-terminal myc epitope. In non-permeabilised human embryonic kidney (HEK) 293T cells transfected with pFLAG-DDX4-myc, positive immunoreactivity was seen using the antibody against the C-terminus of DDX4 (as used by White and colleagues [6]) and an antibody against the myc epitope, consistent with surface expression of the C-terminus of human DDX4. Furthermore, both of these antibodies were used in an antibody-based FACS, on transfected cells, allowing the isolation of DDX4-positive cells, which was confirmed by gene expression.

## 2. Materials and Methods

### 2.1. Immunostaining of Human Tissue

#### 2.1.1. DAB Staining

Human ovarian biopsies were obtained as previously described [21]. To examine human ovarian tissue for the presence of a DDX4-positive cell population, freshly fixed tissue was prepared for immunohistochemistry. Neutral buffered formalin (NBF)-fixed tissue pieces were dehydrated in increasing concentrations of ethanol (70%, 90% and 100%) then placed in cedar wood oil for 24 h British Drug Houses (BDH) Laboratory Supplies, Poole, UK) before clearing with toluene (Fisher Scientific, Loughborough, UK,) for 30 min. Tissue pieces were individually embedded in paraffin wax at 60 °C for 4 h with hourly wax changes, cut into sections of 6 µm, mounted onto slides and left to dry overnight.

**Figure 1:**
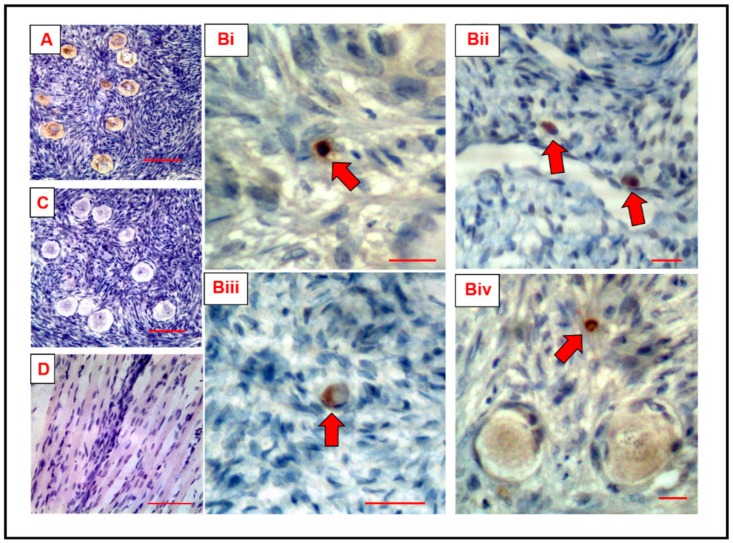
DEAD-box helicase 4 (DDX4) immunohistochemical staining of human ovarian tissue and rat skeletal muscle. (**A**) DDX4-positive (brown staining) germ cells (oocytes) in a section of ovarian cortical tissue from a 23-year-old woman. Scale bar, 100 µm. (**B**) DDX4-positive staining in human ovarian tissue sections from two women aged 31 and 34 years. Small cells staining positive (red arrows) but not associated with oocytes or follicular cells; using Abcam 13840 antibody (i), (i) and (iv) and LS-C97782 antibody (iii). When focusing on (**B**)(iv), it is clear to see the DDX4 staining in the transitory follicles (as expected), in addition to the small putative oogonial stem cell (OSC). Scale bar, 20 µm. (**C**) Negative control: Adult human ovary; primary antibody omitted; no staining of oocytes or other cells. Scale bar, 100 µm. (**D**) Rat skeletal muscle negative control using primary antibody LS-C97782; no staining was visualised. Scale bar, 100 µm; ×40 magnification.

For immunohistochemical detection of DDX4 expression (Figure 1), slides were dewaxed using xylene and decreasing concentrations of ethanol. Antigen retrieval was performed by simmering in 0.01 M sodium citrate for 20 mins and endogenous peroxidases were quenched with 0.3% hydrogen peroxide in methanol. Tissue sections were incubated for an hour in blocking solution (tris-buffered saline (TBS) with 2% normal goat serum (NGS)) then overnight at 4 °C in one of two primary rabbit anti-DDX4 antibodies (ab13840, Abcam, Cambridge, UK or LS-C97782, Lifespan Biosciences, Nottingham, UK) at a concentration of 1 in 500 (ab13840) or 1 in 50 (LS-C97782). Slides were washed repeatedly in TBS-Tween (TBS-T), then incubated for 30 min in a secondary antibody (biotinylated goat anti-rabbit immunoglobulin G (IgG) antibody: 1 in 200) and labelled with horseradish peroxidase (Avidin-Biotin Complex (ABC)-Elite; Vectastain Elite Kit, PK-6101, Vectastain ABC Kit, Vector, Peterborough, UK) for 30 min. DDX4 expression was detected under light microscopy using a 3,3′-diaminobenzidine (DAB) peroxidase substrate kit (Vector Laboratories Ltd., Peterborough, UK). Negative controls included (1) human tissue sections where the primary antibody was omitted and (2) rat skeletal muscle sections stained for DDX4 expression using the primary and secondary antibodies described.

#### 2.1.2. Cell Culture and Transfections, and Generation of the pFLAG-DDX4-myc Construct

**Figure 2:**
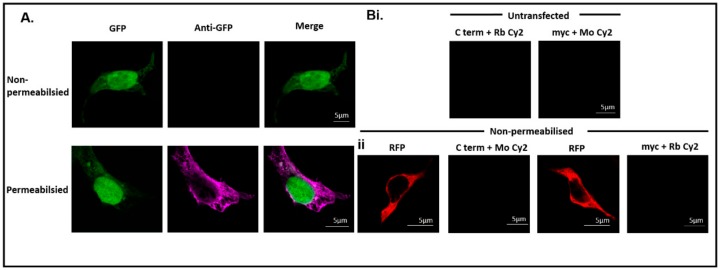
Antibodies do not penetrate non-permeabilised cells. (**A**) Green fluorescent protein (GFP) (green) is visualised in transfected HEK 293T cells. In the non-permeabilised cells fluorescence cannot be detected using an anti-GFP antibody. Fluorescence, from the anti-GFP antibody (magenta), is only detected in permeabilised cells, demonstrating that antibodies do not penetrate non-permeabilised cells. Scale bar, 5 µm. (**B**) (i) Untransfected cells stained with the C-terminus and myc antibodies. No fluorescent signal is detected confirming that there is no non-specific background from the secondary antibodies. (ii) Transfected cells (red) stained with the C-terminus and myc antibodies. The secondary antibodies have been switched, and the lack of fluorescent staining confirmed there was no non-specific binding of the secondary antibodies in non-permeabilised cells. Scale bar, 5 µm.

Cell culture and transfections were performed as previously reported 21. Sequencing results confirmed that full-length DDX4 was successfully cloned into the pDsRed2-C1 vector (refer to Figure S2). Green fluorescent protein (GFP) expression for Figure 2 was achieved using the plasmid pEGFP-C1 (Clontech, Palo Alto, CA, USA).

#### 2.1.3. Double Digests to Confirm Full-Length DDX4 Is Present within the Construct

To ensure that the triple ligation was successful, a series of double restriction, sequential digests were performed on the extracted plasmid DNA. Digests with *BglII* and *XhoI*, *KpnI* and *XhoI* and *AgeI* and *BglII* resulted in isolating the N-terminus (1065 bp), C-terminus (1122 bp) and red fluorescent protein RFP (700 bp) respectively (Figure S3A).

#### 2.1.4. Western Blot

HEK 293T cell homogenates were harvested and heated at 100 °C for 30 min in 2× loading dye (125 mM tris-HCl pH 6.8, 20% glycerol, 4% sodium dodecyl sulphate (SDS), 10% β-mercaptoethanol, 0.25% pyronin Y dye). Protein concentration was determined by comparison to standards using Coomassie Plus Reagent (Thermo Scientific Pierce, Northumberland, UK). Then, 50 µg of protein per sample was loaded onto 4%–20% gradient gels (Life Technologies, Paisley, UK) in tris-glycine/SDS running buffer (25 mM tris-HCl, 52 mM glycine, 0.1% SDS) alongside 5 µL of PageRuler Prestained Protein Ladder (Thermo Scientific, Loughborough, UK) and run at 125 V for 90 min. Proteins were transferred to nitrocellulose membranes (GE Healthcare Life Sciences, Buckinghamshire, UK) in tris-glycine/methanol buffer (25 mM tris-HCl, 192 mM glycine, 20% methanol) for 1 h at 100 V. Membranes were blocked for 1 h at room temperature (RT) in TBS-T supplemented with 2.5% non-fat milk powder before probing with primary antibodies at 4 °C overnight (1:200, anti –(C) DDX4 (Abcam 13840), 1:200, –(N) DDX4 (LS-C109123, 1:200, (Lifespan Biosciences, Seattle, WA, USA), 1:5000, –myc (M4439, Sigma, Dorset, UK), 1:10,000, –FLAG (F1804, Sigma), 1:50,000 and –α-tubulin (179513, Abcam)). The blots were then washed with TBS-T, incubated with either horseradish peroxidase (HRP)-conjugated donkey anti-rabbit IgG or HRP-conjugated sheep anti-mouse IgG (1:5000; Jackson laboratories, West Grove, PA, USA) for 1 h at room temperature before three final washes in TBS-T. Immunoreactive proteins were visualised using enhanced chemiluminescence.

#### 2.1.5. pFLAG-CD2-myc

A vector encoding cluster of differentiation 2 (CD2) was kindly provided by Professor Rose Zamoyska (University of Edinburgh). The aim was to create a pFLAG-CD2-myc construct using the pFLAG-DDX4-myc construct as a backbone. DDX4 would be excised and replaced with CD2. In order to extract the CD2 from its current vector, and DDX4 from pFLAG-DDX4-myc, PCR reactions were performed using a Phusion High-Fidelity PCR Kit (New England Biolabs (NEB), Herts, UK) according to the manufacturer’s instructions. For CD2 excision, the following primers, designed using MacVector 7.2 (Accelrys, Madison, WI, USA), were used: Forward; 5′ TGACGACAAGGGAGTCGACGGAATGAGCTTTCCATGTAAATTTGTA 3′ and reverse 5’ GAGTTTTTGTTCGTCGACTCCATTAGAGGAAGGGGACAATGAGTT 3’. For DDX4 excision, the following primers were used: Forward 5′ GGAGTCGACGAACAAAAACTCATCTCAGAAGAG 3′ and reverse 5′ TCCGTCGACTCCCTTGTCGTCATCGTCTTTG 3′. An initial denaturation step of 96 °C for 3 min was followed by 25 cycles of denaturing at 96 °C for 30 s; annealing at Tm 59 °C for 30 s and extension at 72 °C for 30 s per 500 bp product; with a final extension time of 10 min. Reaction products were treated for 1 h at 37 °C with *DpnI* to remove the original vector backbones. Furthermore, 1.6% agarose gels and a GeneRuler DNA Ladder Mix (Thermo Scientific, Loughborough, UK) were used to analyse the PCR products, which were purified using Zymo DNA Clean and Concentrator (Zymo Research, Irvine, CA, USA). The CD2 primers were designed with 15 bp overhangs so that the CD2 product could be ligated into the cut pFLAG-myc vector by Gibson Assembly (New England Biolabs (NEB), Herts, UK). Following bacterial transformations, DNA extraction was conducted using a QIAprep Spin Miniprep Kit (QIAGEN, Manchester, UK), according to the manufacturer’s instructions.

### 2.2. Immunostaining

#### 2.2.1. Immunofluorescence

Immunocytochemistry was performed as previously described [37]. In brief, cells were washed with 1× phosphate buffered saline (PBS), fixed using 4% paraformaldehyde and permeabilised where appropriate using 0.4% Triton-X 100. Blocking solution (0.2% *w/v* fish skin gelatine (Sigma G-7765) in PBS) was used with or without 0.02% saponin (saponin omitted for non-permeabilised wells). Primary antibodies (mouse anti-c-myc (Sigma), mouse anti-FLAG (Sigma), rabbit anti-DDX4 (ab13840; Abcam), rabbit anti-c-myc (Sigma) and rabbit anti-GFP (A6455, Life Technologies) and secondary antibodies (conjugated goat anti-rabbit Cy2, Cy5 and anti-mouse Cy5, and conjugated donkey anti-mouse Cy2 (Jackson Laboratories, West Grove, PA, USA) were diluted in blocking solution. Dilutions of 1:100–1:1600 were used for the primary antibodies with 1:250 for the secondary antibodies. The cells were incubated in the primary antibodies for an hour, followed by three washes with blocking solution before the secondary antibodies were applied for an hour. Coverslips were then washed three times in blocking buffer and once in water before being mounted with ProLong Diamond Antifade reagent (Thermo Fisher Scientific, Paisley, UK) to prevent photobleaching. Images were captured on a Zeiss LSM510 Axiovert microscope (Jena, Germany) with all acquisition settings kept constant between samples, and colours applied using ImageJ (ImageJ 1.52n, National Institutes of Health, Bethesda, MD, USA).

#### 2.2.2. FACS

HEK 293T cells were transfected with pFLAG-DDX4-myc or FLAG-CD2-myc. Adherent cells were recovered from six-well plates by scraping. Samples were centrifuged at 300× *g* for 5 min, the supernatant was discarded and the cell pellet re-suspended in Hanks’ Balanced Salt Solution (HBSS) with 2% human serum albumin (blocking solution). A small proportion of the cell suspension was removed for unstained and secondary antibody-only controls. The remaining cell suspension was centrifuged at 300× *g* for 5 min, the supernatant was discarded and the cell pellet re-suspended in primary antibody (anti-DDX4 antibody (ab13840, Abcam), mouse anti-myc (Sigma), mouse anti-FLAG (Sigma), rabbit anti-myc (Sigma), dilutions all 1:800) for 40 min. Samples were then centrifuged (300× *g* for 5 min), resuspended in wash buffer (HBSS with 1% HSA) and centrifuged (300× *g* for 5 min) again before being stained with secondary antibodies (conjugated goat anti-rabbit Cy2, anti-mouse Cy5 (Jackson Laboratories, West Grove, PA, USA), dilutions 1:250) for 30 min. The samples were then centrifuged (300× *g* for 5 min) and resuspended in wash buffer. Controls, including untransfected cells, transfected but unstained cells and secondary antibody-only cells, were used to define the gating strategy for cell sorting. The cells were sorted using an Aria II cytometer (Becton, Dickinson and Company (BD) Biosciences, Berkshire, UK) equipped with a 488 nm (to detect Cy2), a 561 nm (to detect (DsRed) and a 633 nm (to detect Cy5) laser. BD FACS DiVa v6 software was used for acquiring data (BD Biosciences, Oxford, UK). Forward scatter and side scatter profiles were used to distinguish intact cells from dead/damaged cells. Flow rate was restricted to 5000 events/s using a 70 µm filter nozzle with a 90 Hz droplet stream. Cells were sorted on high purity and collected into TRIzol reagent (Thermo Fisher Scientific, Paisley, UK) for RT-PCR. Data were analysed and plots generated using FlowJo 9.6v software (FlowJo, LLC, Ashland, OR, USA).

#### 2.2.3. RNA Extraction and RT-PCR

Total RNA was extracted from freshly sorted cells. Cells were lysed by repeated pipetting and incubation in TRIzol reagent. The sample was centrifuged at 12,000× *g* for 10 min at 4 °C then incubated for 5 min at RT. Chloroform was added to the supernatant and eppendorf tubes were shaken vigorously for 15 s before an incubation period of 3 min at RT. The phases were separated by centrifugation (12,000× *g* for 15 min at 4 °C). RNA was precipitated with isopropanol (10 min at RT) and recovered by centrifugation (12,000× *g* for 10 min at 4 °C). The resulting RNA pellet was washed in 75% ethanol, vortexed, then centrifuged (7500× *g* for 5 min at 4 °C), air dried and dissolved in RNase-free water. RNA was quantified spectrophotometrically using a nanodrop; ≥150 ng/µL of RNA was extracted in any experiment. cDNA was generated using an Omniscript Reverse Transcription Kit (Qiagen, Manchester, UK) according to the manufacturer’s instructions; ≤2 µg of RNA was used per cDNA synthesis. PCR was performed using the Phusion High-Fidelity PCR Kit (New England Biolabs), according to the manufacturer’s instructions, with the same conditions as previously mentioned. PCR products were resolved by electrophoresis on a 0.8% TAE agarose gel. Primers used were specifically designed (using MacVector7.2, (Accelrys, Madison, WI, USA)) in the C-terminus of DDX4 (forward 5′ GCTCTTGGAGATTTTCGCTTTGG 3′, reverse 5′ TGCTCTTGCCCTTTCTGGTATC 3′, size 364 bp) and the N-terminus of DDX4 (forward 5′ AGTCAGAAGCAGAAGGAGGAGAAAG 3′, reverse 5′ TGGTAGGAGAAAAGCCGCAGTC 3′, size 353 bp). Negative controls were established by running duplicate reactions omitting the reverse transcriptase enzyme.

## 3. Results

### 3.1. DDX4 Is Expressed in Small Ovarian Cells, in Addition to Human Oocytes

To investigate the specificity of DDX4 localisation in mammalian cells, 6 µm sections of paraffin-embedded adult human ovarian cortex and rat skeletal muscle were probed using two rabbit anti-DDX4 antibodies, ab13840 (Abcam, Cambridge, UK) and LS-C97782 (LifeSpan BioSciences, Inc.), raised against non-overlapping epitopes of DDX4. In adult human ovarian tissue, positive staining was observed as expected in oocytes within follicles at all stages of development using either antibody (Figure 1A). Discrete positive DDX4 staining was also detected in small human ovarian cells (3–15 µm diameter) not associated with follicular structures; this was also observed with both antibodies (Figure 1Bi–iv). These small positively stained cells were frequently detected throughout the ovarian cortex but there was no pattern associated with their localisation. No positive staining was observed in sections of adult human ovary where the primary antibody had been omitted (Figure 1C) or in sections of rat skeletal muscle that had been incubated with primary and secondary antibodies (Figure 1D).

### 3.2. Double-Epitope-Tagged DDX4 Is Expressed in HEK 293T Cells

With the identification of small DDX4-positive non-oocyte ovarian cells, it was imperative to establish whether they could be specifically isolated using antibody-based FACS. To address the issues surrounding the cellular localisation of DDX4, a construct was generated expressing full-length human DDX4 (Figure S2).

HEK 293T cells were transfected with pFLAG-DDX4-myc and immunoblotting was used to determine the molecular weight of the DDX4 protein expressed. The expected molecular weight of full-length DDX4 is 79 kDa, but the RFP tag encoded in pDsRed2-C1 is 28 kDa [38], resulting in the formation of a protein with a molecular weight of approximately 110 kDa. Immunoblotting with antibodies against the C-terminus of DDX4, the myc tag, the N-terminus of DDX4 and the FLAG tag confirmed that full-length DDX4 protein was expressed in transfected HEK 293T cells (Figure S3B). There was no full-length DDX4 protein detected in untransfected cells. This panel of antibodies allowed for complete coverage of DDX4 expression (incorporating both the N- and C-terminus); both fragments of DDX4 and the tags had been successfully cloned into the vector. α-Tubulin confirmed equal protein loading (Figure S3B).

### 3.3. Immunocytochemistry Confirms That the C-Terminus of DDX4 Has an Extracellular Epitope

Green fluorescent protein (GFP) was transfected into HEK 293T cells to ensure the immunocytochemistry procedure did not result in abhorrent staining from inadvertent cell permeabilisation. Immunostaining with an anti-GFP antibody showed no staining in non-permeabilised cells, but clear cytoplasmic staining after the permeabilisation process, confirming no detection of intracellular antigens without permeabilisation (Figure 2A).

There was no fluorescent signal detected in untransfected cells immunostained with the C-terminus antibody (secondary antibody, anti-rabbit Cy2) and the myc antibody (secondary antibody, anti-mouse Cy2, Figure 2Bi) confirming that non-specific binding of the primary and secondary antibodies was not present. To control for non-specific binding of the secondary antibodies only, the secondary antibodies were switched so that anti-mouse Cy2 was incubated with transfected cells treated with the C-terminus antibody and that anti-rabbit Cy2 was incubated will transfected cells treated with the myc antibody. RFP was detected to confirm successful transfection but the fluorophores specific to Cy2 were not detected (Figure 2Bii).

**Figure 3:**
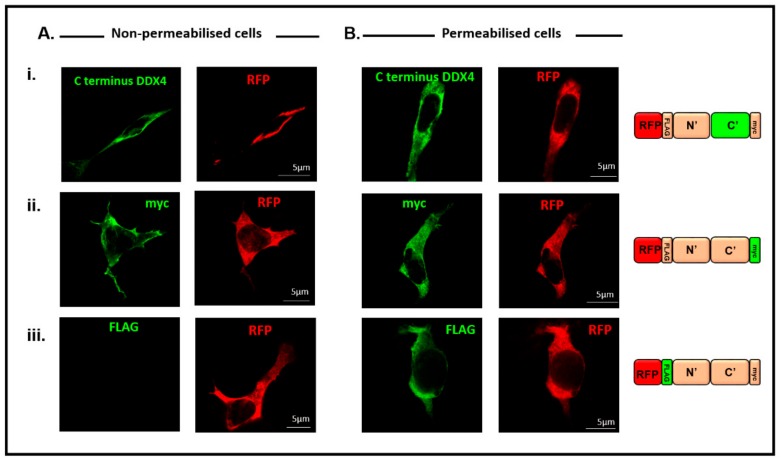
The C-terminus of DDX4 is expressed on the cell surface. (**A**)(i) and (ii) Immunostaining with the C-terminus and myc antibodies (green) confirmed that DDX4 is expressed on the cell surface of transfected (red), non-permeabilised cells. (iii) The majority of non-permeabilised cells confirm that the N-terminus of DDX4 (FLAG tag) is intracellular as the FLAG tag could not be detected in non-permeabilised cells. Red fluorescent protein (red) confirms transfected cells (i), (ii) and (iii). (**B**)(i) and (ii) The C-terminus of DDX4 (green) is observed throughout permeabilised cells. (iii) The N-terminus of DDX4 (FLAG tag, green) can be detected when cells are permeabilised. The colour schemes on the protein schematic mirror the fluorophores used for staining those particular regions of the corresponding protein. Scale bar, 5 µm.

HEK 293T cells were transfected with the pFLAG-DDX4-myc construct and both non-permeabilised (Figure 3A) and permeabilised (Figure 3B) cells were immunostained with antibodies against (i) the C-terminus of DDX4, (ii) the myc tag or (iii) the FLAG tag. RFP was detected in all cells to confirm successful transfection. Figure 3Ai illustrates that the C-terminus of DDX4 is extracellular, as cell surface fluorescence was observed, in non-permeabilised cells, when using an antibody against the C-terminus of DDX4. The same fluorescent pattern was shown by immunostaining with the myc antibody (Figure 3Aii). Fluorescence was not detected in the majority of non-permeabilised transfected cells immunostained with the FLAG antibody (Figure 3Aiii), confirming that the N-terminus of DDX4 is intracellular in these cells. However, 3.5% ± 0.9% of transfected cells did show weak membrane staining of the N-terminus of DDX4 (Figure S4).

Figure 3Bi–iii illustrates that in permeabilised cells, cytoplasmic fluorescence was also observed, indicating that all cellular DDX4 protein was detected, not only that expressed on the cell surface.

**Figure 4:**
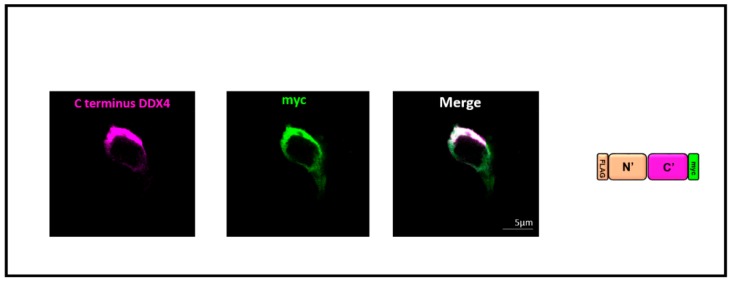
RFP does not affect the cellular localisation of DDX4. RFP was removed from the pFLAG-DDX4-myc construct and when transfected human embryonic kidney (HEK) 293T cells were immunostained with an antibody against the C-terminus of DDX4 (magenta) and the myc tag (green), surface fluorescence was observed. This confirmed that RFP did not affect the cellular localisation of DDX4. The colour schemes on the protein schematic mirror the fluorophores used for staining those particular regions of the corresponding protein. Scale bar, 5 µm.

RFP was removed from the pFLAG-DDX4-myc construct [21] to ensure that RFP was not influencing the cell surface localisation of the fusion protein (Figure 4). When HEK 293T cells were transfected with pFLAG-DDX4-myc (with RFP removed) cell surface staining was detected in non-permeabilised cells when immunostained with both the C-terminus and myc antibodies (Figure 4) confirming that RFP did not affect DDX4 localisation.

### 3.4. DDX4-Positive Cells Isolated by FACS

**Figure 5:**
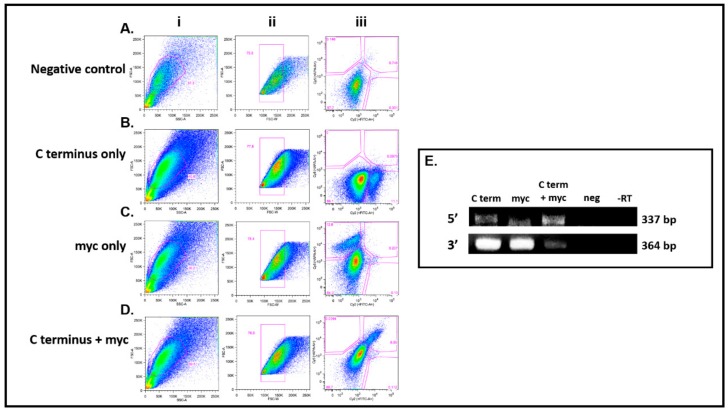
DDX4-positive transfected HEK 293T cells isolated by FACS. Staining of pFLAG-DDX4-myc transfected cells to demonstrate isolation of DDX4 cells using antibodies against the C-terminus of DDX4 or the myc tag. Column (i), intact cell gating (38.5% ± 1%), (ii) intact cells gated to exclude cell aggregates (77.2 ± 0.6%). (**A**)(iii) negative control for non-specific binding of secondary Cy2 and Cy5 antibodies, 97.7% unstained cells. (**B**)(iii) 11.1% positive staining with primary C-terminus and Cy2 secondary antibody. (**C**)(iii) 12.8% positive staining with myc primary and secondary Cy5 antibody. (**D**)(iii**)** 8.55% dual stained using C-terminus and myc antibodies. (**B**iii–**D**iii) Percentage of unstained cells comparable to negative control (86.1, 84.3 and 88.7, respectively). (**E**) 5′ and 3′ *DDX4* gene expression was confirmed in populations sorted using C-terminus antibody alone, myc antibody alone and for dual staining with both. Furthermore, 5′ or 3′ *DDX4* cDNA was not expressed in the double negatives (negative for C-terminus and myc) and reactions where reverse transcriptase was omitted.

To validate the immunocytochemistry results, FACS was performed on transfected HEK 293T cells to determine if DDX4-positive cells could be isolated using an external epitope. Cells were stained with antibodies against (1) the C-terminus of DDX4 (secondary antibody fluorophore Cy2), (2) the myc tag (secondary antibody fluorophore Cy5) and (3) both the C-terminus and myc tag epitopes. Figure 5 shows FACS plots for four different sorts.

Dead and dying cells were identified by applying an electronic gate based on forward and side scatter characteristics, and a secondary gate based on forward scatter area against width was used to exclude cell aggregates before sorting and analysis. Intact cells (Figure 5Ai–Di) were 38.5% ± 1% of the total sample and of these 77.2% ± 0.6% were singlets (Figure 5Aii–Dii). These gates were applied to bivariate fluorescence plots of unstained cells to define sort gates to identify negative, single positive (Cy2 or Cy5) and dual positive populations. Negative controls for non-specific binding of secondary antibodies (Cy2 and Cy5) to transfected HEK 293T showed 97.7% of cells were in the negative gate (Figure 5Aiii). Transfected HEK 293T cells stained with the C-terminus DDX4 antibody alone showed 11.1% were positive (Figure 5Biii). When staining with the myc antibody, 12.8% of cells stained positive (Figure 5Ciii). In transfected HEK 293T cells stained with both the C-terminus antibody and the myc antibody, 8.55%, were in the dual positive sort gate Figure 5Diii). Using both antibodies separately or together, negligible numbers of cells were seen in inappropriate sort gates. The data from the bivariate flow cytometry plots from Figure 5 demonstrate that cells expressing DDX4 can be isolated from non-permeabilised transfected HEK 293T cells using two different antibodies: the C-terminus DDX4 antibody or the myc tag antibody. These data confirm the presence of an external DDX4 epitope.

RT-PCR analysis of 5′ and 3′ *DDX4* gene expression in the sorted cells (Figure 5E) showed that 5′ and 3′ *DDX4* cDNA was detected in positive populations from sorts using the antibodies separately (C-terminus alone or myc alone) and also in the dual positive population when the antibodies were used simultaneously. There was no *DDX4* expression (5′ or 3′) present in the double-negative population and in reactions where reverse transcriptase was omitted (Figure 5E).

### 3.5. The C-Terminus DDX4 Antibody Is Specific to DDX4

To validate the specificity of the DDX4 C-terminus antibody, an additional vector, pFLAG-CD2-myc, was generated. CD2 is a known transmembrane glycoprotein, and its cellular localisation is opposite to that proposed for DDX4 by White et al. (2012). The N-terminus of CD2 is expressed extracellularly whereas the C-terminus is expressed intracellularly [39]. Transfecting HEK 293T cells with pFLAG-CD2-myc and staining for the absent DDX4 external epitope should confirm the specificity and extracellular expression of DDX4 detected using the antibody to the C-terminal epitope.

**Figure 6:**
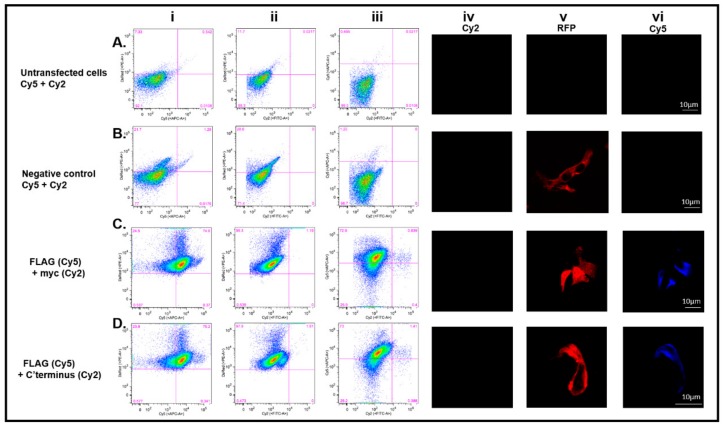
Validation of DDX4 C-terminus antibody specificity using a cluster of differentiation 2 (CD2) transmembrane protein construct. Intact, single cells were gated before analysis and sorting. Column (i)–(iii) (**A**), untransfected cells did not express DsRed and were not stained by secondary Cy5 or Cy2 secondary antibodies (99.5%). Column (i)–(iii) (**B**), transfected cells expressing DsRed (21.7% and 28.6%) did not non-specifically bind secondary antibodies Cy5 or Cy2. (**C**)(i) and (ii) Transfected cells (98.3%) stained with anti-FLAG or anti-myc antibodies were 74.6% Cy5 (FLAG) and 1.19% Cy2 (myc) positive. Dual labelling gave comparable results, FLAG (72.9%) and myc (0.839%) (iii). The results confirm that cells were labelled based on the external epitope (FLAG) but not the internal epitope (myc). Immunostaining confirms that the cells were transfected (v) and that a fluorescent signal could be detected from the FLAG tag (vi) but not the myc tag (iv). Scale bar, 10µm. (**D**) To confirm specificity of the C-terminus DDX4 antibody, CD2 transfected cells were immunostained for the FLAG tag (Cy5) and the C-terminus of DDX4 (Cy2). Of 97.9% DsRed-positive cells, 75.2% were labelled with Cy5 only (i) and only 1.61% was labelled with Cy2 (ii). These results confirm that the FLAG epitope could be detected but that there was no positive signal associated with the C-terminus antibody. Immunostaining confirms that the cells were transfected (v) and that the FLAG tag could be detected (vi) but there was no positive signal emitted from the C-terminus antibody (iv). Scale bar, 10 µm.

As expected, cells transfected with pFLAG-CD2-myc were detected in the DsRed-positive gate. Transfected cells were stained with a FLAG antibody (Cy5) and the myc antibody (Cy2) to determine that non-permeabilised CD2-positive cells were sorted based on expression of the external FLAG tag, and not the internal myc tag. Cells were sorted after gating for intact, single cells (Figure 6). Untransfected HEK 293T cells were negative for DsRed expression and, when stained with secondary Cy5 or Cy2 antibody alone (Figure 6Ai,ii), showed 92.1% and 88.3% respectively. Moreover, 99.5% of cells were negative when Cy5 and Cy2 were used together (Figure 6Aiii). Immunofluorescence (Figure 6Aiv–vi) confirmed that there was no non-specific binding of secondary antibodies to untransfected cells Cy2 (Figure 6Aiv) or Cy5 (Figure 6Avi).

The negative controls using transfected HEK 293T cells also showed no non-specific binding of Cy5 or Cy2 secondary antibodies alone (Figure 6Bi-ii, respectively) or together (Figure 6Biii). 21.7% (Figure 6Bi) and 28.6% (Figure 6Bii) were DsRed-positive, but 98.7% of cells were negative for both Cy5 and Cy2 (Figure 6Biii). This was confirmed by immunofluorescent studies (Figure 6Biv–vi).

Sorting of transfected DsRed CD2-positive cells (98.3% +ve) using the external FLAG tag showed 74.6% co-expressed FLAG (Cy5 +ve) (Figure 6Ci), whereas the same cells were completely negative for internally expressed myc (Cy2 –ve) (Figure 6Cii). Dual staining showed 72.9% FLAG +ve cells and 0.4% myc +ve (Figure 6Ciii). These results were supported by immunostaining. Non-permeabilised DsRed +ve cells (Figure 6Cv) stained positive for FLAG (Figure 6Cvi) but not for myc (Figure 6Civ).

To test the specificity of the C-terminus DDX4 antibody, the CD2 transfected HEK 293T cells were dual labelled with the C-terminus DDX4 and FLAG antibodies (Figure 6D). It was found that 97.9% of intact cells were DsRed positive but negative for C-terminus DDX4 (Cy2) (Figure 6Dii), and 75.2% were dual labelled with DsRed and FLAG (Cy5) (Figure 6Di). Immunocytochemistry confirmed that transfected non-permeabilised cells were positive for DsRed (Figure 6Dv) and FLAG (Cy5) (Figure 6Dvi) but negative for C-terminus DDX4 (Cy2) (Figure 6Div). These results demonstrate that when CD2-positive cells are incubated with the DDX4 C-terminus antibody, there is no positive staining confirming the specificity of the C-terminus antibody for the DDX4 protein and that external detection of the DDX4 C-terminus seen in DDX4 transfected HEK cells was valid.

## 4. Discussion

Isolating putative OSCs from adult ovarian tissue using the cell surface expression of the germline marker DDX4 is controversial [36,40,41,42,43,44,45,46]. While putative OSCs have been isolated using Ddx4/DDX4 and a FACS-based protocol [6,21], scepticism persists about the specificity of DDX4 antibodies and whether Ddx4/DDX4 is naturally expressed on the cell surface.

In this study, we have identified that a population of small cells (size range 3–15 µm) exists in adult human ovarian cortex, consistent with reports of the isolation of such cells from the human ovary [6,21] that are not oocytes and yet stain positively for DDX4 using two different DDX4 antibodies, whose non-overlapping epitopes are in the C-terminus. Before pursuing further characterisation of these cells, it is essential to first establish if the C-terminus of DDX4 can be extracellular as proposed by White et al. [6].

We designed a DDX4 expression construct, to investigate cellular localisation of this RNA helicase, using N- and C-terminal epitope tags. FLAG and myc tags allowed distinct detection of each terminus of DDX4, removing reliance on the C-terminus DDX4 antibody.

When saponin was omitted from the reaction, antibodies against an N-terminal FLAG epitope were unable to detect DDX4 expression in HEK 293T cells. Under the same conditions, antibodies against a C-terminal myc epitope and the C-terminal specific DDX4 antibody were able to detect DDX4, consistent with a cell surface expression of the C-terminus of DDX4, as reported previously in putative OSCs using the same C-terminus DDX4 antibody [21]. We confirmed that in the absence of saponin, antibodies were unable to detect intracellular epitopes. These results are contrary to results obtained by Hernandez et al. [26], who labelled the C-terminus of DDX4 with a myc tag but could not detect any myc expression in non-permeabilised human ovarian cells, with a weak fluorescent signal observed in the permeabilised cells. This difference may reflect differences in expression levels following lentiviral transfection rather than the plasmid-based mammalian vector used in this study. It should be noted, however, that the immunostaining we detect does not always co-localise completely with the red fluorescent fusion protein, which may indicate that epitopes associated with DDX4 can have variable antibody accessibility (Figure 3). This may explain some differences seen in previous studies based on DDX4 immunodetection [26,35,36].

Clearly HEK 293T cells are not OSCs but are being used here as a model system for proof of principle. These cells may possess protein-trafficking or post-translational modification systems distinct from those present in stem cells. However, these results demonstrate that contrary to predictions from the primary structure of human DDX4 expressed in stem cells, the protein can be transported to the surface of a heterologous cell type.

In order to further validate the results obtained from immunocytochemistry, we tested if transfected cells could be sorted by antibody-based fluorescence. FACS was used to identify human cells transfected with pFLAG-DDX4-myc. DDX4-positive populations were obtained with antibodies against either the C-terminus of DDX4 or its myc tag, and it was confirmed that these sorted cells also expressed *DDX4* cDNA. The same primer sets were used that confirmed *DDX4* cDNA expression in human putative OSCs [21].

To ensure that the FACS process was able to isolate cells based on antibodies binding to external epitopes and not to intracellular domains, cells were transfected with a construct encoding for the transmembrane protein, CD2. CD2 was cloned into our vector so that the protein had differential tags expressed at the N (FLAG) and C (myc) terminus. Intact transfected cells could be isolated with antibodies against the extracellular FLAG tag, but not with antibodies against the myc tag, consistent with an intracellular localisation. The results from this experiment validated the use of FACS to isolate cells based on extracellular protein domains.

In summary, we have shown that the C-terminus of DDX4 is present on the surface of transfected mammalian cells and can be used to sort DDX4-positive cell populations. The antibody used to confirm the extracellular localisation of the C-terminus of pFLAG-DDX4-myc, also identified a population of cells within the adult human ovary, which may be those identified by White et al. [6] and others [15,16,17,18,19,20,21]. Therefore, the use of FACS with antibody-based DDX4 detection is an effective method for OSC isolation [6] and supports other studies that use this method for putative OSC detection and isolation. Despite criticisms of using DDX4 to isolate OSCs, positive DDX4 populations have been seen to form follicle-like structures in vitro [4,5,6,7,8,14,16,17,19,20,21,25] and in vivo [6,14]. The challenge now is to fully characterise the populations of cells within the adult mammalian ovary and to determine the conditions that might support new oocyte formation in vitro and in vivo.

## Supplementary Materials

The following are available online at https://www.mdpi.com/2073-4409/8/6/578/s1, Figure S1: The contentious localisation of DDX4 in putative mammalian OSCs. The traditional model on the left, shows the RNA helicase DDX4 being present in its area of function; the cytoplasm. The FACS model on the right shows that a portion of the DDX4 protein is extracellular. White *et al*. utilised an antibody that binds to a purported epitope within this extracellular C-terminus for FACS isolation of DDX4 positive cells (Adapted from Linder and Jankowsky (2011) [47]; [6]). Figure S2: Sequence of pFLAG-DDX4-myc. The RFP sequence is highlighted in red; the FLAG tag sequence is highlighted in purple; the restriction sites are highlighted in blue and the myc tag sequence is highlighted in yellow. The DDX4 sequence is represented by lower case letters. Samples were sequenced by the Human Genetics Unit, Western General Hospital, Edinburgh, and analysed with A Plasmid Editor (aPE) software. Figure S3: Restriction digests confirmed that full length human DDX4 had been cloned into pDsRed2-C1. A: DNA cut with *BglII* and *XhoI* (lane 1), separates the N terminal fragment (~1000 bp) from the cut vector (~5000 bp). Bands of ~1000 bp (C-terminus) and ~ 5000 bp (cut vector) were also seen in lane 2 when DNA was cut with *KpnI* and *XhoI*. In order to identify the RFP (~700 bp), the vector (~6000 bp) was cut with *AgeI* and *BglII* (lane 3). B: HEK 293T cells, transfected with pFLAG-DDX4-myc, express full length DDX4. Western blot analysis confirmed that full length (110KDa) DDX4 protein was detected when antibodies against epitopes in both the N-(FLAG and N DDX4) and C-(myc and C DDX4) terminus of DDX4 were used. No protein was detected in the untransfected cells. α-tubulin was used as a protein loading control and was detected in all loaded samples. Figure S4: Cellular localisation of the N-terminus of DDX4. Weak cell surface expression of the FLAG tag (green) in a transfected HEK 293T cell (red). The colour schemes on the protein schematic mirror the fluorophores used for staining those particular regions of the corresponding protein. Scale bar, 5 µm.
