# Supplementary material for: Extracellular Localisation of the C-Terminus of DDX4 Confirmed by Immunocytochemistry and Fluorescence-Activated Cell Sorting
Source: Cells. 2019 Jun 12;8(6):578. doi: 10.3390/cells8060578 (PMC6627596; doi:10.3390/cells8060578)
Supplement: Supplementary file 1 [file cells-08-00578-s001.pdf]

Supplementary Information

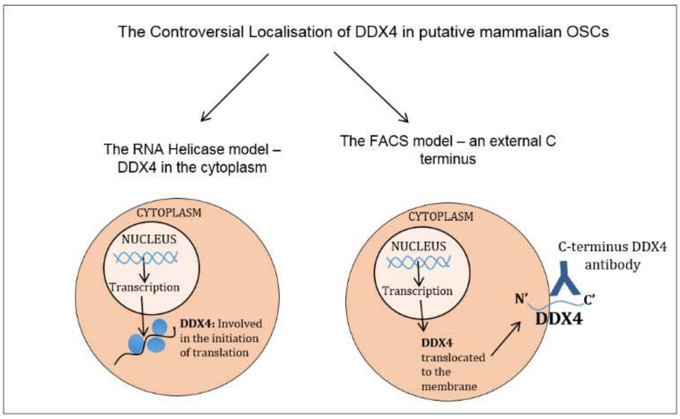

Figure S1: The contentious localisation of DDX4 in putative mammalian OSCs. The traditional model on the left, shows the RNA helicase DDX4 being present in its area of function; the cytoplasm. The FACS model on the right shows that a portion of the DDX4 protein is extracellular. White *et al*. utilised an antibody that binds to a purported epitope within this extracellular C-terminus for FACS isolation of DDX4 positive cells (Adapted from Linder and Jankowsky (2011) [47] ; [6]).

Sequencing of pFLAG-DDX4-myc

Key:
RFP
FLAG tag
BglII (AGATCT), *XhoI* (ctcgag), *KpnI* (GGTACC) – restriction sites
Lower case letters = DDX4 sequence
Myc tag

ATGNCGNAACAACTCCGCCCCATTGACGCAAATGGGCGGTAGGCGTGTACGGTGGNNNNTNNANA
TAAGCAGAGCTGGTTTAGTGAACCGTCAGATCCGCTAGCGCTACCGGTCGCCACCATGGCCTCCTCC
GAGAACGTCATCACCGAGTTCATGCGCTTCAAGGTGCGCATGGAGGGCACCGTGAACGGCCACGAG
TTCGAGATCGAGGGCGAGGGCGAGGGCCGCCCCTACGAGGGCCACAACACCGTGAAGCTGAAGGT
GACCAAGGGCGGCCCCCTGCCCTTCGCCTGGGACATCCTGTCCCCCCAGTTCCAGTACGGCTCCAAG
GTGTACGTGAAGCACCCCGCCGACATCCCCGACTACAAGAAGCTGTCCTTCCCCGAGGGCTTCAAGT
GGGAGCGCGTGATGAACTTCGAGGACGGCGGCGTGGCGACCGTGACCCAGGACTCCTCCCTGCAG
GACGGCTGCTTCATCTACAAGGTGAAGTTCATCGGCGTGAACTTCCCCTCCGACGGCCCCGTGATGC
AGAAGAAGACCATGGGCTGGGAGGCCTCCACCGAGCGCCTGTACCCCCGCGACGGCCCCGNTGNN
GCANAACAANACCATGGGCNGGGAGGCCTCCNCNGANCGCCTGTNCCNCCNNNACGGCGTGCTG
AAGGGACGGCGTGCTGAAGGGCNAGACCCACNAGGCCCTGNAGCTGAAGGACGGCGGCCACTAC
CTGGNGGAGTTCAAGTCCNTCTACNTGGCCNNNAAGCCCGTGCAGCTGCCCGGCTACTACTACNTG
GACNCCAAGCTGGACNTCACCTCCCACAACGAGGACTACNCCATCGTGGAGCANTACNAGCGCACC
GAGGGCCGCCACCACCTGTTCCTGAGATCTATGGACTACAAAGACGATGACGACAAGatggagacaatt
ttaacaggactccagcttcatcatcagaaatggatgatggaccttctcgaagagatcatttcatgaaaagtggatttgcctctgggcg
gaattttggaaacagagatgctggtgagtgtaataagcgagataatacatccacaatgggtggttttggagttggaaagagttttgg
aaacagaggtttttcaaacagcaggtttgaagatggtgatagctctggtttctggagagagtctagtaatgactgcgaagataatcc
aacacggaacagagggttttccaagagaggcggctatcgagatggaaataattcagaagcttcagggccatacagaagaggtgg
aagaggtagtttccgaggttgccgtggaggatttggtctaggaagtccaaataatgacttagacccagacgaatgtatgcagcgca
ctggtggcctttttggttctagaagaccagtattaagtggcacaggtaatggtgatacttctcaaagcagaagtggcagtggaagtg
aacgaggtggttacaaaggtttaaatgaagaagtaataacaggctctggaaagaattcttggaagtcagaagcagaaggaggag
aaagtagtgatactcaaggaccaaaagtgacctacatacccccctcctccacctgaggatgaggactccatctttgcacattatcaga
caggcataaacttcgacaaatacgacactattcttgtggaagtgtctggacatgatgcaccaccagcaattctgacttttgaagaag
ctaatctctgtcagacactgaataacaacattgctaaagctggttatactaagcttactcctgtgcaaaaatacagtattcctatcata
cttgcaggacgagatttgatggcttgcgctcaaacagggtctgggaagactgcggcttttctcctaccaattttggctcatatgatgca
tgatggaataactgccagtcgttttaaagagttgcaggaaccagagtgtattattgtagcaccaactcgagaattggtcaaccagat
ttatttggaagccagaaaattttcttttgggacttgtgtaagagctgttgttatatatgggggaacccagctgggacattcaattcgac
aaatagtacaaggctgtaatatattatgtgctactcctggaagactgatggatatcataggcaaagaaaagattggtctcaaacag
atcaaatacttagttttggatgaagctgatcgcatgttggatatgggttttggtccagaaatgaagaagttaatttcttgcccaggaat
gccatcaaaggaacagcgccaaacccttatgttcagtgcaacttttccagaggaaattcaaaggttggctgcagagtttttaaagtc
aaattatctgtttgttgctgttggacaagtgggtggagcatgtagagatgttcagcagaccgttctccaagttggccagttctcaaaa
agagaaaagctcgttgaaattctgcgaaacataggggatgaaagaactatggtctttgttgaaactaagaaaaaagcagattttat
tgcaacttttctttgtcaagaaaaaatatcaactacaagtattcatggtgatcgggaacagagagagcgggagcaagctcttggag
attttcgctttggaaagtgcccagttcttgttgctacttcagtagctgccagagggctggatattgaaaatgtgcaacatgttatcaatt
ttgatcttccttctaccattgatgaatatgttcatcgaattgggcgtactggtcgttgtgggaatactggcagagcaatttcctttttga
tcttgaatcggataaccatttagcacagcctctagtaaaagtattgacagatgctcaacaggatgttcctgcatggttggaagaaatt
gcctttagtacatacattcctggcttcagtggtagtacaagaggaaacgtgtttgcatcagttgataccagaaagggcaagagcact
ttgaacacagctgggttttcttcttcacaagctcccaatccagtagatgatgagtcatgggatCTGGTCCCCCCGCGGCAGC
AAAGAACAAAAACTCATCTCAGAAGAGGATCTGAAATAAGGTACCGCGGGCCCGGG

Figure S2: Sequence of pFLAG-DDX4-myc. The RFP sequence is highlighted in red; the FLAG tag sequence is highlighted in purple; the restriction sites are highlighted in blue and the myc tag sequence is highlighted in yellow. The DDX4 sequence is represented by lower case letters. Samples were sequenced by the Human Genetics Unit, Western General Hospital, Edinburgh, and analysed with A Plasmid Editor (aPE) software.

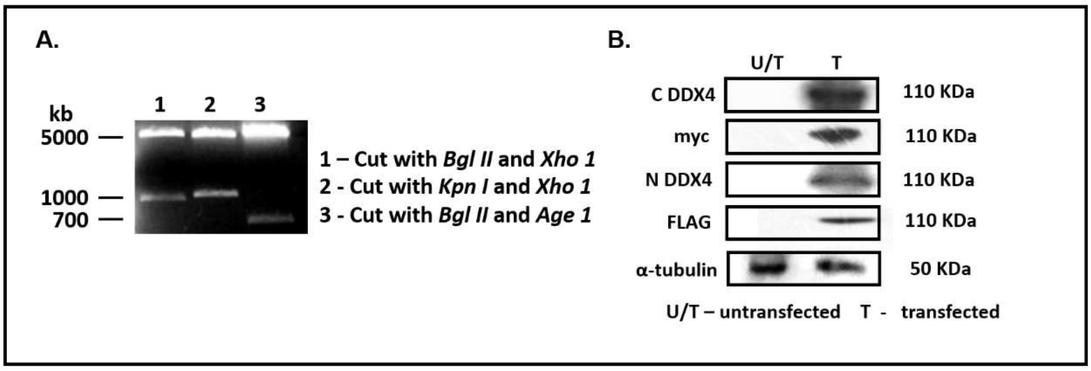

Figure S3: Restriction digests confirmed that full length human DDX4 had been cloned into pDsRed2-C1. A: DNA cut with *BglII* and *XhoI* (lane 1), separates the N terminal fragment (~1000bp) from the cut vector (~5000bp). Bands of ~1000bp (C-terminus) and ~ 5000 bp (cut vector) were also seen in lane 2 when DNA was cut with *KpnI* and *XhoI*. In order to identify the RFP (~700bp), the vector (~6000bp) was cut with *AgeI* and *BglII* (lane 3). **B:** HEK 293T cells, transfected with pFLAG-DDX4-myc, express full length DDX4. Western blot analysis confirmed that full length (110KDa) DDX4 protein was detected when antibodies against epitopes in both the N-(FLAG and N DDX4) and C-(myc and C DDX4) terminus of DDX4 were used. No protein was detected in the untransfected cells. α-tubulin was used as a protein loading control and was detected in all loaded samples.

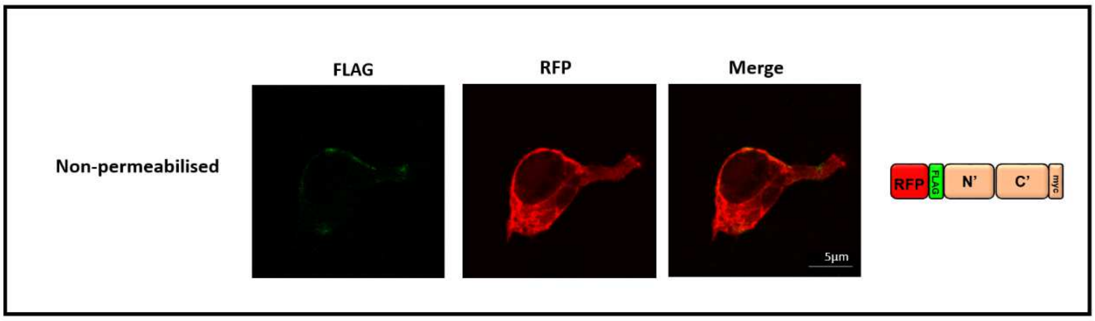

Figure S4: Cellular localisation of the N-terminus of DDX4. Weak cell surface expression of the FLAG tag (green) in a transfected HEK 293T cell (red). The colour schemes on the protein schematic mirror the fluorophores used for staining those particular regions of the corresponding protein. Scale bar, 5µm.
